# One year of digital teaching in psychiatry as a response to the COVID-19 pandemic: Knowledge gain and content evaluation of medical students for two summer semesters in 2020 and 2021

**DOI:** 10.1371/journal.pone.0276660

**Published:** 2022-10-21

**Authors:** Matthias Besse, Jörg Signerski-Krieger, Jens Wiltfang, Claudia Bartels, Michael Belz

**Affiliations:** 1 Department of Psychiatry and Psychotherapy, University Medical Center Göttingen, Göttingen, Lower-Saxony, Germany; 2 German Center for Neurodegenerative Diseases (DZNE), Goettingen, Germany; 3 Department of Medical Sciences, Neurosciences and Signaling Group, Institute of Biomedicine (iBiMED), University of Aveiro, Aveiro, Portugal; King Abdulaziz University Faculty of Medicine, SAUDI ARABIA

## Abstract

After the beginning of the COVID-19 pandemic in 2020, digital teaching had to be implemented by most universities at short notice and widely replaced classroom teaching. As a consequence, digital teaching further reduced direct social interaction for students. One year after the introduction of digital teaching formats at our university medical center (department of psychiatry and psychotherapy), teaching evaluation of students from summer semesters 2020 and 2021 (SS20, SS21) were compared. The main objective of this study was to objectify whether students evaluate digital teaching less favorably after one year of its implementation. Ratings of 311 medical students on (1) knowledge gain, (2) teaching contents and (3) subjective advantages of digital teaching were analyzed for the two separate cohorts SS20 (*n* = 175) and SS21 (*n* = 136). Students also rated their pandemic-related stress level, and if learning progress had been reduced by the pandemic in general. Significant knowledge gain was achieved for all included domains in psychiatry (all *p* < .001), and did not differ between SS20/SS21. Teaching contents in SS21 were rated worse in six out of eight domains compared to SS20 (*p* < .001 to .05). Also, subjective advantages of digital teaching vanished in most domains comparing the cohorts of SS21 and SS20 (*p* < .001 to .05). No differences were found for pandemic-related stress level and subjective learning progress. Limitations include the post-hoc design, possible bias from individual exam grades, and sampling bias. The present study showed that knowledge gain can be considered to be stable one year after the pandemic-related implementation of digital teaching. However, sustainability of this teaching format should be monitored critically: The subject of psychiatry and psychotherapy thrives on direct communication, which can be compromised when using digital formats only. In this light, implementation of more interactive formats in digital teaching is discussed.

## Introduction

The COVID-19 pandemic has posed major challenges for the German university landscape in light of the pandemic-related measures as defined by the Infection Protection Act [1]. For the first time, it was decided for the summer semester 2020 that instead of previously established classroom teaching, all university courses had to be offered in digital form in order to maintain teaching operations. Contrary to initial hopes, the prolonged course of the COVID-19 pandemic [2] prevented a timely return to classroom teaching. For this reason, digital teaching had to be carried forward to the 2020/2021 winter semester and the 2021 summer semester at German universities [3].

Besides these structural challenges, each individual student faced various limitations in both personal and professional contexts as a result of the COVID-19 pandemic (e.g., fewer patient contacts). Several studies found that the pandemic had a negative impact on students’ mental health and general well-being [4–7]. The significant reduction of direct social interaction in distance teaching formats like digital teaching [8] may have played a pivotal role here, which, in addition to the restrictions in general social life, still represents a relevant stress factor for many students until today [9, 10].

In principle, digital teaching formats such as lecture podcasts were already available before the COVID-19 pandemic, and had shown to achieve a comparable learning success in earlier studies [11, 12]. However, they had not been widely implemented across all universities and disciplines [13]. Due to the pandemic, the switch to digital teaching had to take place within a few weeks at most universities [14–17]. As in other universities [18], teachers at the University Medical Center Goettingen (UMG) initially were concerned that the lack of direct interaction with students could lead to a decline in the quality of teaching. Particularly for the subject of psychiatry and psychotherapy, which thrives on direct and intensive communication as well as social skills, the question arose as to whether the inevitable digitization could achieve similar learning success as classroom teaching.

In their study, Besse et al. [19] showed that the initial implementation of digital teaching in psychiatry as a response to the COVID-19 pandemic did not entail a decrease in knowledge gain or a worse evaluation of teaching contents when compared to classroom teaching. On the contrary, their findings underlined that–in 2020 –knowledge gain and evaluation were at least on an equal or even superior level under digital teaching conditions when compared to earlier semesters with classroom teaching, although at the cost of a higher workload for students. However, as the pandemic has continued for another year, it is unclear if these effects still hold up to this point: As multiple studies suggest, students persistently experienced social isolation from the beginning of the pandemic (see above). Since then, they have been continuously confronted with digital teaching to an extent never seen before. The study presented here has the main objective to answer if there is a saturation effect emerging: It aims to objectify whether students have become less positive about digital teaching after one year of its implementation, compared to a separate evaluation during the first introduction of this teaching format. This study extends the findings from Besse et al. [19]: There, medical students evaluated the newly introduced digital teaching format, exclusively for the summer semester 2020. New data are now available for the current study, which compares the teaching evaluations of 311 medical students at the Department of Psychiatry and Psychotherapy, UMG, for the years 2020 and 2021 with a focus on (1) knowledge gain, (2) teaching contents, and (3) subjective advantages of digital teaching. To our knowledge, this study is the first to directly compare undergraduate medical teaching in psychiatry during the COVID-19 pandemic for two consecutive summer semesters.

## Materials and methods

### Study and sample design

A total of *N* = 311 medical students in their 5^th^ clinical semester were included in the present study. Students belonged to one of two subgroups (1) summer semester 2020 (SS20, participation *n* = 175 out of 179, 97.8%; Ø exam grade 1.8, Ø exam difficulty 0.84) or (2) summer semester 2021 (SS21, participation *n* = 136 out of 155, 87.7%; Ø exam grade 2.3, Ø exam difficulty 0.81). The participation rate in SS21 was significantly lower than in SS20 (χ^2^(1, *N* = 334) = 13.01, *p* < .001). The survey was conducted as a post-hoc paper-and-pencil questionnaire, carried out at the end of the respective semester (SS20 or SS21). The EvaSys system (Electric Paper Evaluation Systems) was used to scan and import the completed questionnaires (please see S1 File for study data). Since the survey was part of the standardized teaching evaluation at the UMG, no demographic data (gender, age, etc.) or individual exam results were allowed to be collected for the present sample in order to preserve anonymity of the students. For this reason, an evaluation of the existing free-text comments was not carried out either. The paper-and-pencil questionnaires were completed by the students immediately after their module exam. All questionnaires were distributed in advance on the individual exam workplaces. Therefore, all students who wrote the mandatory module exam had the opportunity to participate in the study. As the data was collected voluntarily, including no personal information and/or patient data, obtaining informed consent was not required. Since the study presented here was not subject to either the German Medicines Act or the Medical Devices Act, no ethics application had to be submitted to the Ethics Committee of the University Medical Center Göttingen.

### Implementation of digital teaching in psychiatry: 2020 and 2021

#### Lectures

The initial implementation of digital teaching took place in SS20 (please see Table 1 for a comparison between SS20/SS21 vs. classroom teaching pre-SS20). In lectures, students were taught the theoretical basis of psychiatric disorders (including the psychiatric examination) and their pharmacological and psychotherapeutic treatment. The lectures were digitally recorded in the form of podcasts and made available to the students on the university’s online platform Stud.IP (GPL) for the entire duration of the semester. The program Camtasia (TechSmith) was used to record the lectures, enabling the lecturers to record the PowerPoint presentations together with spoken audio. All lectures were limited to a duration of 30 up to 45 minutes at the most. For SS21, all but two podcasts were re-recorded with Camtasia Studio optimizing the recording quality and the actuality of the lectures. In SS21, a “digital consultation hour” related to the lectures was offered three times a week as an additional exchange platform. Here, the lecturers of the previous days were available to the students in a live conference room for questions about the respective podcasts.

**Table 1:** Comparison of teaching formats: Classroom teaching vs. digital teaching.

*Classroom teaching (before SS20)*	*Digital teaching SS20*	*Digital teaching SS21*
**Lectures**		
Traditional lecture in the lecture hall:	Podcasts of lectures:	Podcasts of the lectures (new recordings)^1^:
• Patient presentation in the context of the lecture	• Example video with acting patient	• Example video with acting patient
• Direct interaction (e.g. questions) in the lecture	• Indirect interaction (e.g. questions) via forum	• Indirect interaction via forum and direct interaction in “digital consultation hours”
**Seminars**		
Seminars in small groups:	Inverted-classroom concept:	Inverted-classroom concept:
• Short theoretical input by the lecturer	• Short podcast with theoretical background	• Short podcast with theoretical background
• Followed by collective working on cases/exploration of patients within each topic	• Working tasks on an individual basis	• Working tasks on an individual basis (reduced amount of tasks)^2^
	• Feedback from lecturers	• Feedback from lecturers
**Bedside teaching**		
• Psychiatric examination of patients in face-to-face contact (small student groups) • Discussion of observations • Direct interaction (e.g. questions) with the lecturer	• same as for classroom teaching	• same as for classroom teaching

^1^All but two podcasts were newly recorded for *SS21* (summer semester 2021).

^2^The number of cases to be worked on was reduced from three to two, due to the workload reported by students in *SS20* (summer semester 2020).

#### Seminars

For the seminars, a podcast lasting a maximum of 15 minutes was recorded for each of three seminar sections (see below), in which the students received a short theoretical input on each topic. Following the podcast, the students were given tasks that they worked on independently. This corresponds in principle to the concept of the “inverted classroom”, which is also used in the context of classroom events to actively promote independent learning [20].

In the seminar section (1) “psychopathological assessment”, various psychiatric disorders were portrayed with the help of professional actors and recorded as a video podcast. The task of the students of SS20 was to write a brief medical report including the presented psychopathology for three cases. Due to the high workload reported by these students, the number of cases to be worked on was reduced to two instead of three for SS21. Both in SS20 and SS21, the students in the seminar section (2) “psychiatric emergencies” had to process two psychiatric emergency situations presented in writing and suggest concrete procedures in the respective situation. Through the seminar podcast, the basic steps in psychiatric emergency situations were explained in advance based on three emergency situations. Also, the seminar section (3) “stigmatization” was identical for SS20 and SS21: After a short theoretical input via podcast, a simulated doctor-patient interaction was presented, whereby the students were asked to identify and describe the characteristics for stigmatization occurring in the conversation.

The written seminar tasks were sent by e-mail to the lecturers. The students also received individual feedback by e-mail. The forum integrated in Stud.IP was used for communication between students and lecturers both in SS20 and SS21. Students could use it to ask questions about the content of courses or organizational procedures during the semester.

#### Bedside teaching

Bedside teaching was carried out in both semesters face-to-face and in compliance with hygienic regulations. However, in SS20 it could not take place at the end of the module as planned, but at a later time-point at the end of the semester after the first wave of the pandemic had subsided.

### Study measures

The questionnaire used in this study contained 24 items for the primary outcomes, out of which 22 were formulated as statements and could be answered via 6-stage scales (1 = “fully applies” to 6 = “does not apply”). Please refer to S1 Table for all item formulations. Additionally, students were asked to award an overall school grade for the whole teaching module (1 = “very good” to 6 = “insufficient”).

#### Knowledge gain

Measurement of knowledge gain was realized with paired items as pre-established at the UMG. The items contained seven statements for selected objectives in psychiatry (e.g. “I know the different types of dementia”), each to be rated on a 6-stage scale (see above). Students retrospectively rated their subjective pre-teaching and their current i.e. post-teaching knowledge level in the domains of (1) psychopathological assessment, (2) pharmacological treatment of attention deficit hyperactivity disorder (ADHD), (3) psychotherapeutic interventions, (4) types of dementia, (5) antidepressants, (6) schizophrenia, (7) anxiety disorders. Students were additionally asked to estimate their weekly time expenditure in hours, both for the entire module (neurology, child and adolescent psychiatry, psychosomatics, psychiatry) and for the subject psychiatry alone. Both workloads in hours were used as control variables (see S1 Table).

#### Teaching contents

Students evaluated the mediation of teaching contents in the subject psychiatry within the teaching module via eight items, each formulated as a statement (e.g. “In terms of my professional future, I rate my knowledge gain in psychiatry in this module as very high.”). These items are used by default to evaluate the teaching at the UMG. All items were rated with the 6-stage scale, covering the following areas: (1) implementation of interdisciplinary teaching, (2) promotion of independent learning (autonomy), (3) knowledge gain related to professional future, (4) satisfaction with the basic structure of the module, (5) practical implementation of teaching, (6) preference to continue the module in this form, (7) subjective learning progress through lectures, (8) subjective learning progress through seminars.

#### Subjective advantages of digital teaching

Students were asked to rate subjective advantages of digital teaching. These items were used for the first time during SS20, after the teaching formats reported above (Table 1) had been implemented (see also Besse et al. [19]), and were again deployed in SS21. Five statements made specific assumptions about digital teaching (e.g. “My knowledge gain in the subject of psychiatry is higher with digital teaching compared to classroom teaching.”), and could then be rated by students on the 6-stage scale: (1) higher knowledge gain, (2) preference for digital teaching, (3) more workload, (4) better preparation for the written state exam, (5) better preparation for a medical profession in the future. Two control items were also implemented, comprising (a) a possible reduction of learning progress and (b) an increased stress level, both in relation to the COVID-19 pandemic (see S1 Table).

### Statistical analyses

Data were analyzed using SPSS®, version 27. Means (*M*), standard deviations (±), and Pearson correlations (*r*) were calculated for descriptive presentation. Statistical tests were conducted for three primary outcomes of this study. (1) For the assessment of *knowledge gain*, seven general linear models for repeated measurement (GLM) were used. Within each model, retrospective (pre-teaching) and current self-assessments (post-teaching) were added as a two-staged within-subject factor. Furthermore, the semester (SS20 vs. SS21) was added as a two-staged between-subjects factor. In addition, test statistics for the interaction of the two factors were calculated for each GLM to identify possible differences in knowledge gain trajectories between the two semesters (self-assessment × semester).

Multiple *t*-tests for independent samples (semester: SS20 vs. SS21) were calculated to analyze differences in (2) the ratings on *teaching content* between both semesters (8 items) and (3) *subjective advantages of digital teaching* over classroom teaching (7 items). Additionally, differences in weekly workload were analyzed between both semesters (2 items; see S1 Table).

Due to α-error inflation, *p*-values were adjusted according to the Bonferroni method for the number of 24 statistical tests in total (*p*_*adj*_ = *p*_*emp*_ × 24). Based on this level of correction, an additional adjustment was made for all reported pairwise comparisons within each GLM. In individual cases, students provided incomplete information in the questionnaires by omitting items–see the degrees of freedom for each model or statistical test for included cases (mean completion rate for all items within the total sample: 88.1%). Completion rates between SS20 (86.4%) and SS21 (90.4%) differed by 4.1%.

Besides our main sample, we also included ratings from three previous semesters who exclusively participated in pre-pandemic classroom teaching: (1) WS18/19 (*n* = 67, participation rate 45.6%, Ø exam grade 2.8), (2) SS19 (*n* = 58, participation rate 40.8%, Ø exam grade 2.1), and (3) WS 19/20 (*n* = 50, participation rate 35.2%, Ø exam grade 2.0; please see Besse et al. [19] for details). These students had answered the same items on *teaching content* as their fellow students did in SS20/SS21. Here, their ratings were used as reference values for visual comparison (see results section for details), but were not included into inferential statistical analysis.

## Results

### Knowledge gain

A knowledge gain from pre- to post-teaching ratings was found for all seven assessed domains within the total sample (see Table 2, deltas from *M* = -1.79 to -2.77). This increase was highly significant with large effect sizes for all domains, ranging from “(b) pharmacological treatment of ADHD” (*F*(1, 287) = 475.48, *p* < .001, partial η^2^ = 0.62) to “(a) psychopathological assessment” (*F*(1, 283) = 1250.75, *p* < .001, partial η^2^ = 0.82). Increasing weekly working hours spent for learning correlated with increased knowledge gain, reaching significance for “(b) pharmacological treatment of ADHD”, “(f) schizophrenia”, and (g) “anxiety disorders” (*r* = -.161 to -.168, *p* .011 to .017, see Table 2).

**Table 2:** Knowledge gain (SS20 vs. SS21).

Variable	1	2	3	4	5	6	7	8	9	*M* ± SD / freq.
1. Semester (SS20 vs. SS21)	–									SS20: 175, SS21: 136
2. Working hours: module	-.113	–								36.12 ± 10.59
3. Working hours: psychiatry	-.173**	.646**	–							25.42 ± 11.29
4. Δ Psychopathology	.053	-.088	-.100	–						-2.27 ± 1.33
5. Δ Pharmacology ADHD	.070	-.021	-.161*	.388**	–					-1.79 ± 1.38
6. Δ Psychotherapy	-.067	-.074	-.110	.404**	.465**	–				-2.34 ± 1.40
7. Δ Dementia	.072	-.065	-.082	.294**	.426**	.411**	–			-2.49 ± 1.27
8. Δ Antidepressants	.037	-.078	-.123	.333**	.366**	.400**	.460**	–		-2.13 ± 1.15
9. Δ Schizophrenia	.067	-.081	-.165*	.327**	.467**	.521**	.540**	.501**	–	-2.77 ± 1.33
10. Δ Anxiety disorders	.082	-.063	-.168*	.368**	.458**	.485**	.502**	.465**	.664**	-2.52 ± 1.22

Correlations

*p < .05

**p < .01; *M* = mean value, ± *SD* = standard deviation, *freq*. = frequency. *SS20* = summer semester 2020 (coded as “1”, *n* = 175), *SS21* = summer semester 2021 (coded as “2”, *n* = 136); *working hours* in hours per week; Δ items 4 to 10: delta of the subjectively rated knowledge gain from pre-teaching to post teaching for seven domains in the field of psychiatry (Δ = (post-teaching)—(pre-teaching)), negative values indicate a knowledge gain from pre- to post-teaching (*N* = 311).

**Fig 1:**
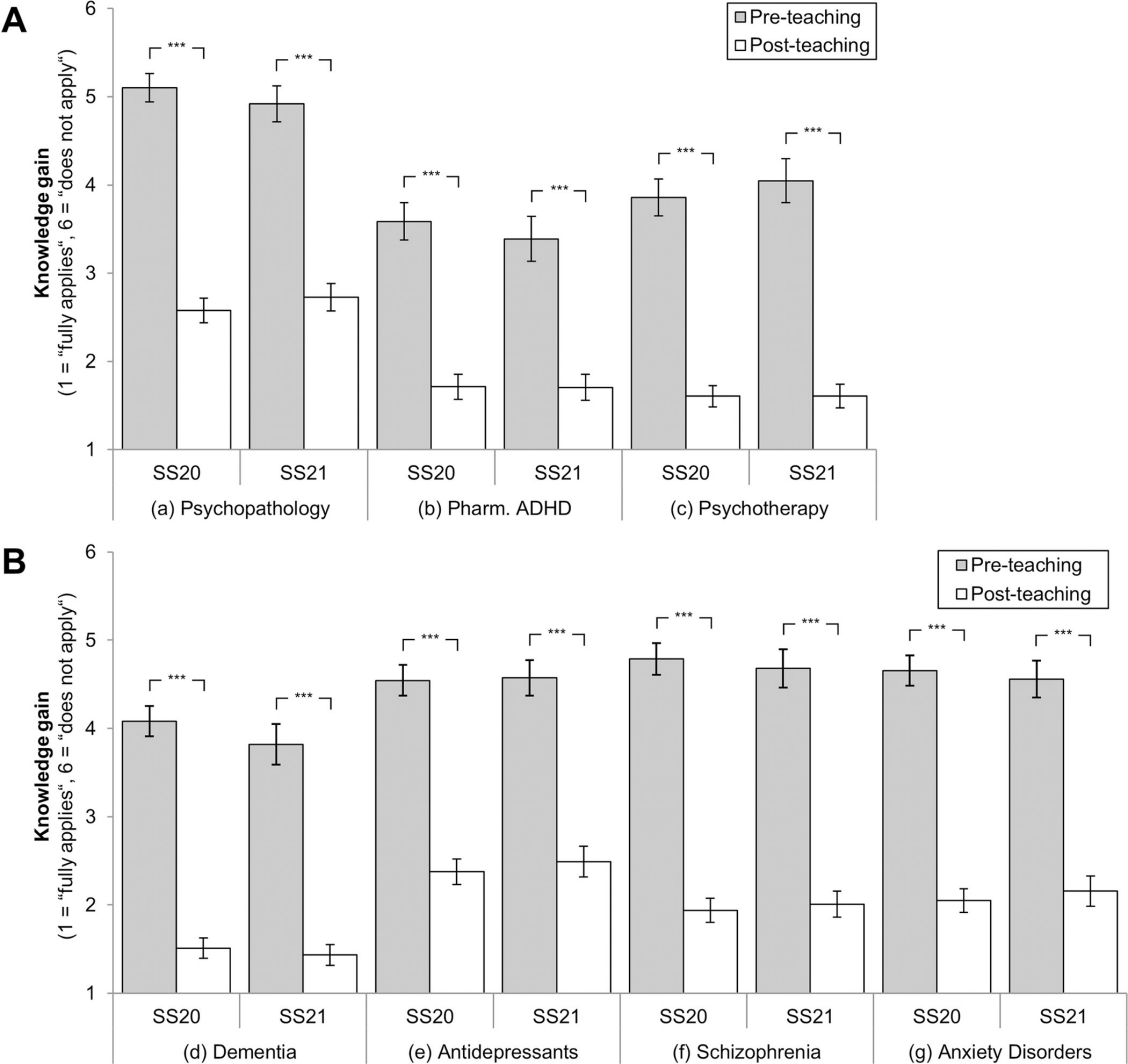
Knowledge gain in digital teaching, as rated by students in SS20 (*n* = 175) and SS21 (*n* = 136). * *p* < 0.05, ** *p* < 0.01, *** *p* < 0.001. Mean values with 95%-CIs and Bonferroni corrected pairwise comparisons. Students rated statements retrospectively (pre-teaching) and post-teaching on a 6-stage scale (1 = “fully applies”, 6 = “does not apply”), e.g., “I know the different types of dementia” (please see S1 Table for all item formulations). *N* = 311.

When analyzed separately, both SS20 and SS21 showed comparable knowledge gains for all domains (see Fig 1A and 1B): Deltas for SS20 ranged from *M* = -1.88 to *M* = -2.85, deltas for SS21 ranged from *M* = -1.68 to *M* = -2.67. For both semesters, all pairwise comparisons revealed a significant improvement from retrospective to current self-assessment (pre- to post-teaching ratings: all *p* < .001, see Fig 1A and 1B). Correlations additionally showed that students tended to invest slightly less weekly working hours for learning in SS21 if compared to SS20 (see Table 2), but pairwise comparisons did not reveal significant differences, neither for working hours spent for the whole module nor for teaching in psychiatry alone (*t* = 1.79 to 2.69, *p* = .181 to .999).

Besides the knowledge gain reported above, GLMs did not reveal between-subjects effects (SS20 vs. SS21)–the general level of knowledge did not differ between both semesters in any of the seven domains (GLMs: *F* < .01 to 3.51, *ns*). Furthermore, analyses did not reveal any significant interaction effects (GLMs: *F* = 0.39 to 6.23, *ns*): The trajectories of knowledge gain from pre- to post-teaching did thus not systematically differ between both semesters (see Fig 1A and 1B). In sum, the knowledge gain can be considered as being statistically equivalent between both semesters in all assessed domains.

### Teaching contents

**Fig 2:**
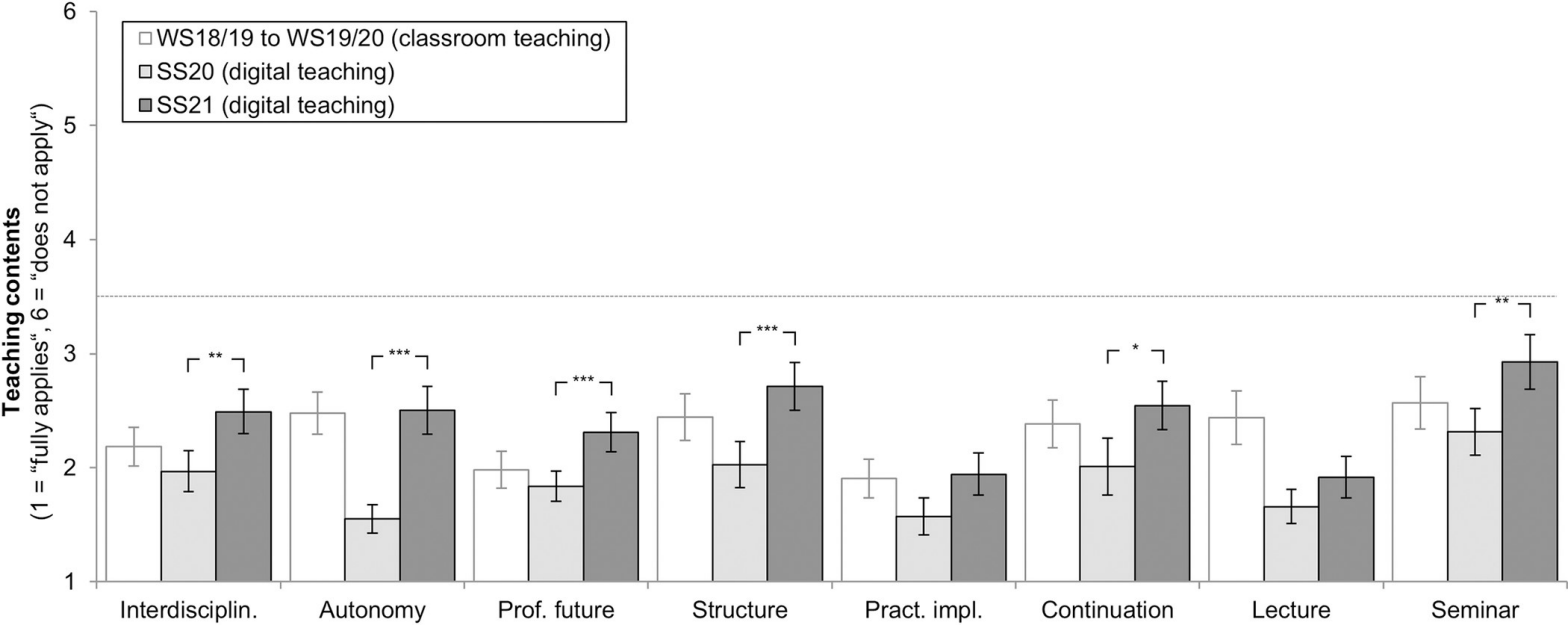
Teaching contents rated by students for SS20 (*n* = 175) and SS21 (*n* = 136). * *p* < 0.05, ** *p* < 0.01, *** *p* < 0.001. Mean values with 95%-CIs and Bonferroni corrected pairwise comparisons. Students rated statements on a 6-stage scale (1 = “fully applies”, 6 = “does not apply”), e.g., “This module should be continued as is with respect to the subject of psychiatry” (please see S1 Table for all item formulations): (1) implementation of interdisciplinary teaching, (2) promotion of independent learning (autonomy), (3) knowledge gain related to professional future, (4) satisfaction with the basic structure of the module, (5) practical implementation of teaching, (6) preference to continue the module in this form, (7) subjective learning progress through lectures, (8) subjective learning progress through seminars. Ratings from semesters WS2018/19 to WS2019/20 (classroom teaching) were added as reference values for visual comparison. *N* = 311 (digital teaching), *n* = 175 (classroom teaching).

For SS20, students awarded a mean school grade of 1.86 for teaching in psychiatry (between “very good” and “good”). For SS21, this rating resulted in a mean grade of 2.31 (between “good” and “satisfactory”). Regarding evaluation of teaching contents, students from SS21 gave numerically worse ratings for all eight items when compared to SS20. Significance was reached for six out of eight items (see Fig 2): (1) implementation of interdisciplinary teaching (*t*(238) = -3.89, *p* = .003), (2) promotion of independent learning (*t*(279) = -8.01, *p* < .001), (3) knowledge gain related to professional future (*t*(290) = -4.30, *p* < .001), (4) satisfaction with the basic structure of the module (*t*(276) = -4.62, *p* < .001), (5) preference to continue the module in this form (*t*(226) = -3.23, *p* = .034), (6) subjective learning progress through seminars (*t*(267) = -3.85, *p* = .004). Despite this majority of poorer ratings, values for SS21 did not exceed the neutral value of 3.5 on the scale for any item (minimum value: *M* = 1.92 ± 1.01 maximum value: *M* = 2.93 ± 1.34, see Fig 2).

#### Explorative comparison with pre-pandemic classroom teaching

To enable an exploratory and more precise classification of these findings, ratings from students who had participated in classroom teaching from 2018 to 2020 (winter semester (WS) 18/19 to winter semester 19/20, *n* = 175, see Besse et al. [19] for details) were also integrated in Fig 2 as reference values. In relation to SS21, differences were smaller–still, students in SS21 gave numerically worse ratings than students having participated in classroom teaching (WS18/19 to WS19/20) in seven out of eight domains. One exception was the perceived contribution of lectures to learning progress: For this item, students in SS21 gave better ratings (*M* = 1.92 ± 1.01) than students in classroom teaching (*M* = 2.43 ± 1.39, see Fig 2).

### Subjective advantages of digital teaching

**Fig 3:**
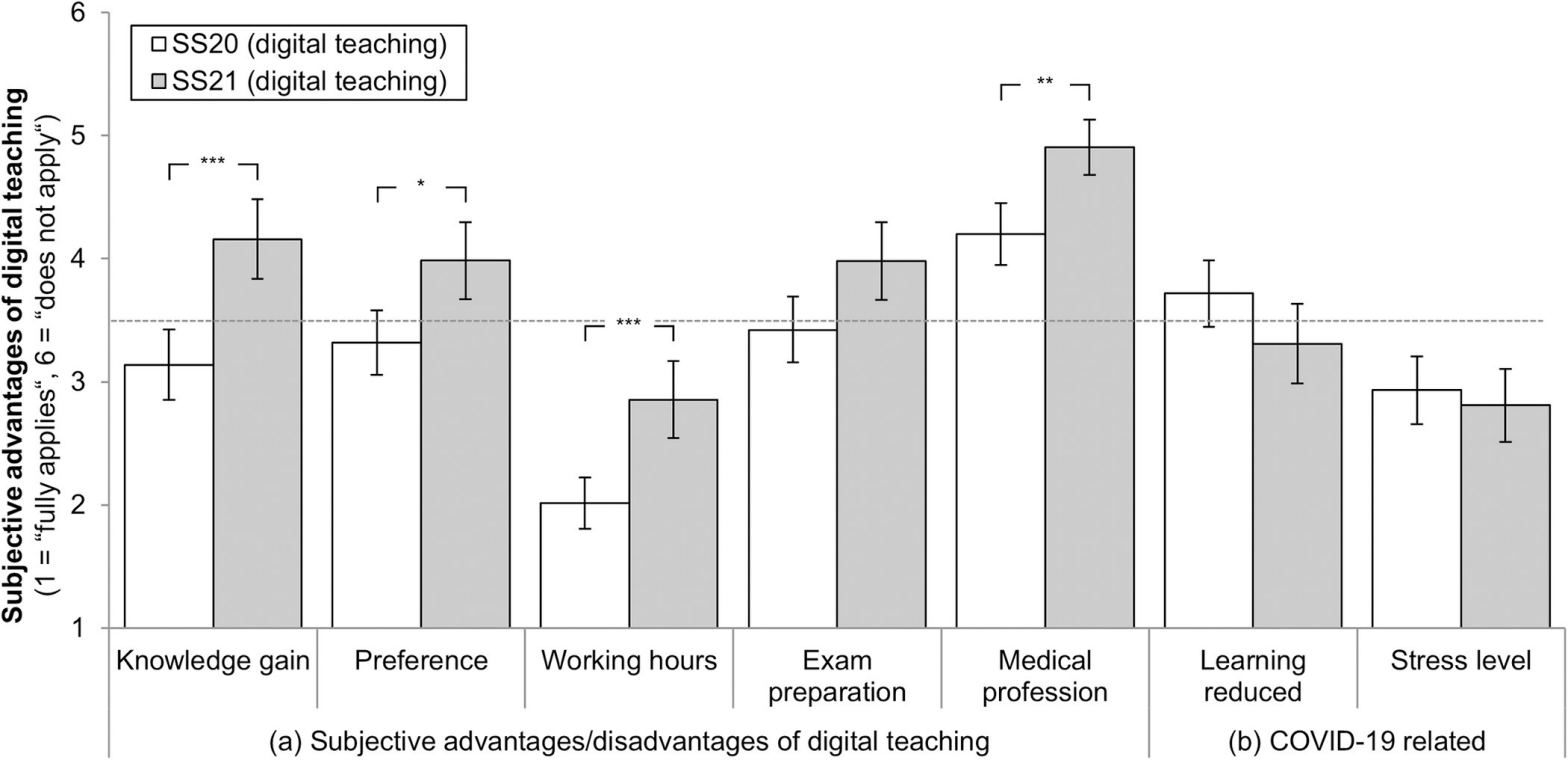
Subjective advantages of digital teaching as rated by students in SS20 (*n* = 175) and SS21 (*n* = 136). * *p* < 0.05, ** *p* < 0.01, *** *p* < 0.001. Mean values with 95%-CIs and Bonferroni corrected pairwise comparisons. Students rated statements on a 6-stage scale (1 = “fully applies”, 6 = “does not apply”), e.g., “My knowledge gain in the subject of psychiatry is greater with digital teaching than in classroom teaching” (please see S1 Table for all item formulations): (1) better knowledge gain, (2) preference for digital teaching, (3) more working hours (oder workload), (4) better preparation for the written state exam, (5) better preparation for a medical profession in the future, (6) related to the COVID-19 pandemic: reduction of learning success, (7) related to the COVID-19 pandemic: higher stress level. *N* = 311.

In line with the teaching contents, students from SS21 rated potential advantages of digital teaching worse in three out of five items when compared to SS20. Significant differences in favor of SS20 were found for (1) higher knowledge gain (*t*(245) = -4.63, *p* < .001), (2) preference for digital teaching (*t*(277) = -3.22, *p* = .034), (3) better preparation for a medical profession in the future (*t*(253) = -4.01, *p* = .002). In comparison to SS20, students from SS21 agreed to a lesser extent to the statement that digital teaching generally leads to a higher workload (*t*(282) = -4.58, *p* < .001). In sum, values for SS21 exceeded the neutral value of 3.5 on the scale for four items (*M* = 3.98 ± 1.79 to *M* = 4.90 ± 1.22, see Fig 3). Concerning a possible influence of the COVID-19 pandemic, analyses did not reveal any significant differences in terms of a reduction in subjective learning progress (*t*(262) = 1.91, *p* = .999) or an increased stress level (*t*(287) = 0.61, *p* = .999) between both semesters (see Fig 3).

## Discussion

The present study investigated whether digital teaching in psychiatry–ad hoc and widely realized with the onset of the COVID-19 pandemic in 2020 –results in a satisfactory teaching evaluation by undergraduate medical students after more than a year of its implementation. For this purpose, the ratings of 311 medical students on (1) knowledge gain, (2) teaching contents, and (3) subjective advantages of digital teaching were analyzed for two consecutive semesters in summer 2020 and 2021. It was found that knowledge gain remained stable in SS21. Mediation of teaching contents and subjective advantages of digital teaching were both rated worse in SS21 compared to SS20.

It is remarkable that knowledge gain during SS21 was both equivalent to SS20 as well as to the knowledge gain of pre-pandemic semesters with face-to-face teaching (see Besse et al. for details [19]). This is in line with other studies, which found comparable knowledge gain / teaching success between digital- and face-to-face-teaching [11, 12]. Our study additionally underlined that knowledge gain achieved with digital teaching potentially remains stable at a level comparable to classroom teaching even more than one year after its initial implementation. This is of particular interest considering that students simultaneously rated digital teaching worse in the other domains examined here. Especially subjective advantages of digital teaching were not only rated worse in SS21, but exceeded the neutral value of 3.5 in the negative range for four out of five items. Furthermore, the mediation of teaching contents was rated worse in SS21, albeit not as low as subjective advantages. In sum, students seemed to clearly differentiate between their subjective, satisfactory knowledge gain achieved by this teaching format, but were coevally less enthusiastic about its positive aspects after one year of implementation. It is hard to identify possible reasons for this discrepancy: In contrast to our findings, several studies showed that students rather appreciate time flexibility in digital teaching [21–23]. There are other characteristics of this teaching format, which are generally valued by students, like determining the own learning pace or accessibility [24, 25]. However, there are some studies which indicate that initial satisfaction with digital teaching formats implemented due to the COVID-19 pandemic do not remain permanent, e.g., as reported by physicians from different fields: There, these formats led to frustration due to high number and/or frequency [26]. In sum, longitudinal studies are needed to analyze changes on students’ evaluations of digital teaching, and to better understand the discrepancy between a satisfying knowledge gain and a more negative view on other aspects of digital teaching found here.

Several studies have now been published that describe the COVID-19-related transfer of teaching into digital formats specifically in the discipline of psychiatry. However, the focus of these studies lies on the detailed description of the newly established digital teaching formats and the associated challenges [27–29]. Statements on knowledge gain or the general evaluation of digital teaching from the students’ point of view can only be found sporadically and in few studies: Morris et al. were able to show that after the digitization of psychiatric teaching at their university, students felt well prepared with regard to examinations and the following practical forms of teaching [30]. In an online survey of students at eight medical schools in Sri Lanka, students rated digital lecture formats in psychiatry as an effective teaching method [31]. A survey of 24 responsible persons for psychiatric teaching at medical schools in the United Kingdom also indicates a high level of satisfaction with digital teaching in psychiatry on the part of students [32]. To our knowledge, our study is the only one to evaluate knowledge gain, teaching contents and subjective advantages of this teaching format in psychiatry for two consecutive summer semesters.

The subjective burden of the COVID-19 pandemic on the individual learning progress and the associated stress level potentially bias teaching evaluations. Both were not rated higher in SS21 compared to SS20 and should therefore not have played a major role here–although multiple other studies clearly found increased stress levels in students during the continuing pandemic [4–7, 33]. Interestingly, students in SS21 stated to generally invest less time in digital teaching than the students in SS20. In line with this, the weekly mean working time of 4.7 hours for the module was rated to be lower than in SS20 (5.4 hours). The poorer rating of digital teaching is thus by no means due to a further increase in workload as working tasks had been reduced in SS21. Additionally, the lower workload might be seen as a result of the increasing experience of the students with digital teaching. It can also be assumed that the final module exam could have been a potential factor influencing the overall teaching evaluation, which was written after the theoretical/teaching part of the module. The results of the exam in SS21 were worse with an average grade of 2.3 compared to SS20 (Ø 1.8). Also the average difficulty in SS21 was higher with 0.8099 compared to the SS20 (Ø 0.8439). It is well known that achieved exam grades correlate with the students’ evaluation of previous teaching, and accordingly, worse exam grades lead to worse teaching evaluations [34]. Since in our case the evaluation took place immediately after the module exam, this might have had a relevant influence on the present teaching evaluation. One additional factor which could have influenced students’ evaluation is the motivation of teaching personnel, which potentially declined during the pandemic [35]. In our case, the same teaching personnel were used both during SS20 and SS21 which would at least eliminate a selection bias. However, the motivation of these lectures could still have suffered from SS20 to SS21. This may be especially important for psychiatric teaching, which at least implicitly thrives on direct, non-digital interaction between teaching personnel and students. Future studies should therefore analyze both the students’ as well as the lecturers’ perspective in this matter to cover this hitherto insufficiently researched area. The aim should be to clarify if a possible saturation effect and loss of motivation due to digital teaching formats specifically applies to the field of psychiatry. Moreover, the way students work may also have changed during the pandemic, e.g., by using online meeting learning platforms [36]. This was not mapped in our survey and should be focused on in future studies.

In contrast to SS20, students in SS21 had significantly more experience with digital teaching formats. It is conceivable that students had become acquainted with digital techniques in previous digital semesters, which had not previously been integrated into our module and were now anticipated as obligatory–live interaction between students and lecturers in seminars can be mentioned as one example. Consistent with this assumption, the partially podcast-based seminars in SS21 were rated significantly worse for the same content and teaching format, but the digital lecture podcasts were not. In other modules of the clinical study section at our university hospital, more seminars have been progressively conducted as live video streaming with the introduction of digital teaching. For teaching in psychiatry, this was exclusively realized in the context of the “digital consultation hours”. Lectures continued to take place in almost every module in the form of recorded podcasts. Although this is not directly evident from our study, it is reasonable to assume that live digital seminars are preferred by students, while lectures are more likely to be accepted in form of podcasts. So far, studies could show a high degree of satisfaction with live digital teaching methods, even in comparison to face-to-face teaching [37, 38]. It also seems that the most intensive and direct communication possible between teachers and students is associated with greater learning satisfaction [39, 40]. In sum, this underlines the necessity of continuously evaluating digital teaching and adapting it to the preferences and needs of students.

Such an adaptation seems to be imperative, especially with regard to later professional life. Both in SS20 and SS21, students indicated that they tended to feel less prepared for later medical profession by digital teaching–this has also been found in other studies [41–43]. In this context, Alsoufi et al. found that a majority of students considered e-learning as being inadequate for conveying clinical aspects [44]. Digital teaching, no matter how innovative it may be, seems to reach its limits in this discipline. It may successfully sustain contact between students and patients [45], but cannot fully replace direct contact in a clinical context. This may especially be the case in the field of psychiatry and psychotherapy. Here, this was the main reason to carry out the bedside teaching face-to-face.

There are some limitations to this study. First, knowledge gain in this study was measured post hoc. In a self-assessment, students both rated their subjective knowledge pre- and post-teaching, realizing pre-teaching assessments as retrospective ratings. Such an approach is potentially more prone to measurement error and results must always be interpreted with caution. Overall, a longitudinal design would be preferable, but would require two separate measurements (pre- and post-teaching) for each individual. This was not feasible in our case due to strict requirement for complete anonymity of all data collected as part of the teaching evaluation: Each finalized questionnaire had to be handled as a stand-alone anonymous data source. It should also be noted that two minor, feedback-based changes have been made between SS20 and SS21: (1) the “digital consultation hour” was implemented as additional discussion platform between lecturers and students, (2) working tasks for seminars were reduced from three (SS20) to two (SS21). However, the majority of teaching content was not changed (see Table 1). Nevertheless, this might have biased students’ evaluations between SS20 and SS21. On the other hand, the teaching personnel did not change at all between both semesters, thus improving the comparability of both semesters. Second, individual exam grades would represent an objective operationalization of learning progress, which could be used in addition to judge (a) the performance of teaching and (b) the validity of self-assessed knowledge gain. Again, due to strict anonymity requirements, the assignment of individual exam results to the individual teaching evaluation was not possible. In line with that, a detailed sample description (age, gender, etc.) would also be preferable to control for systematic differences between SS20 and SS21, but as this information was not collected during teaching evaluation to guarantee the students’ anonymity, systematic differences between biographical subgroups could not be analyzed. Third, although all students who took the module exam were given the opportunity to participate in the survey, a sampling bias cannot be completely ruled out: The participation rate during SS21 (87.7%) was significantly lower, if compared to SS20 (97.8%). It has thus to be assumed that the student population was less accurately represented one year after the implementation of digital teaching, although the overall participation rate can be considered high. At this point, one can only speculate about the reasons for the lower level of participation during SS21. In addition to a possible saturation effect which may have led to less motivation to evaluate digital teaching, the experienced higher exam difficulty and worse exam grades in SS21 may also have reduced the students’ participation rate: As the evaluation took place directly after each module exam it is quite conceivable that motivation for the subsequent evaluation might have been affected negatively. Fourth, generalization of our results to other disciplines is limited per se, as this study exclusively focused on the field of psychiatry.

Since the onset of the COVID-19 pandemic, universities have made significant efforts to successfully enable teaching with digital formats in the short term. While the initial implementation of this teaching format in 2020 was potentially perceived as the only way to sustain university teaching and thus was positively received by students, the present study shows that the level of knowledge gain in 2021 sustained even one year after the initial introduction of digital teaching in 2020. However, it also casts at least slight doubts on the full sustainability of this teaching format: Here, even improvements of digital teaching in 2021 could not prevent poorer evaluation results in several domains. As a consequence, students must be continuously involved in the development of digital teaching in order to consider their needs. It remains to be seen whether pandemic-related measures as social restrictions will cause increasingly negative perceptions towards digitization and, subsequently, digital teaching formats in the future. A constant further development of digital teaching is essential, as it will be an important part of teaching in the future, also in the field of psychiatry.

## Supporting information

S1 Table Questionnaire items. English translation of all 24 questionnaire items and their response scales which were used in this study.
(DOCX)

S1 File Dataset of the study. The dataset of the study is provided as SAV-file (SPSS).
(SAV)

## References

[1] Federal Parliament Germany. Fourth Law for the Protection of the Population in the Event of an Epidemic Situation of National Significance. Apr 22, 2021 pp. 1–6. Available: http://www.bgbl.de/xaver/bgbl/start.xav?startbk=Bundesanzeiger_BGBl&jumpTo=bgbl121s0802.pdf

[2] Federal Ministry of Health Germany. Coronavirus pandemic (SARS-CoV-2): chronicle of actions and events to date. 2021. Available: https://www.bundesgesundheitsministerium.de/coronavirus/chronik-coronavirus.html

[3] German Rectors’ Conference. Requirements and regulations of the federal states for teaching and examination at universities during the Corona pandemic. 2021. Available: https://www.hrk.de/themen/hochschulsystem/covid-19-pandemie-und-die-hochschulen/

[4] Son C, Hegde S, Smith A, Wang X, Sasangohar F. Effects of COVID-19 on College Students’ Mental Health in the United States: Interview Survey Study. J Med Internet Res. 2020;22: e21279. doi: 10.2196/21279 32805704 PMC7473764

[5] Wang X, Hegde S, Son C, Keller B, Smith A, Sasangohar F. Investigating Mental Health of US College Students During the COVID-19 Pandemic: Cross-Sectional Survey Study. J Med Internet Res. 2020;22: e22817. doi: 10.2196/22817 32897868 PMC7505693

[6] Meda N, Pardini S, Slongo I, Bodini L, Zordan MA, Rigobello P, et al. Students’ mental health problems before, during, and after COVID-19 lockdown in Italy. J Psychiatr Res. 2021;134: 69–77. doi: 10.1016/j.jpsychires.2020.12.045 33360865

[7] Dodd RH, Dadaczynski K, Okan O, McCaffery KJ, Pickles K. Psychological Wellbeing and Academic Experience of University Students in Australia during COVID-19. Int J Environ Res Public Health. 2021;18: 866. doi: 10.3390/ijerph18030866 33498376 PMC7908219

[8] Chaturvedi K, Vishwakarma DK, Singh N. COVID-19 and its impact on education, social life and mental health of students: A survey. Children and Youth Services Review. 2021;121: 105866. doi: 10.1016/j.childyouth.2020.105866 33390636 PMC7762625

[9] Elmer T, Mepham K, Stadtfeld C. Students under lockdown: Comparisons of students’ social networks and mental health before and during the COVID-19 crisis in Switzerland. PLoS One. 2020;15: e0236337. doi: 10.1371/journal.pone.0236337 32702065 PMC7377438

[10] Hamza CA, Ewing L, Heath NL, Goldstein AL. When social isolation is nothing new: A longitudinal study on psychological distress during COVID-19 among university students with and without preexisting mental health concerns. Can Psychol. 2021;62: 20–30. doi: 10.1037/cap0000255

[11] Schreiber BE, Fukuta J, Gordon F. Live lecture versus video podcast in undergraduate medical education: A randomised controlled trial. BMC Med Educ. 2010;10: 68. doi: 10.1186/1472-6920-10-68 20932302 PMC2958969

[12] Pei L, Wu H. Does online learning work better than offline learning in undergraduate medical education? A systematic review and meta-analysis. Med Educ Online. 2019;24: 1666538. doi: 10.1080/10872981.2019.1666538 31526248 PMC6758693

[13] Kuhn S, Frankenhauser S, Tolks D. [Digital learning and teaching in medical education: Already there or still at the beginning? Bundesgesundheitsblatt Gesundheitsforschung Gesundheitsschutz. 2018;61: 201–209. doi: 10.1007/s00103-017-2673-z 29234823

[14] Skulmowski A, Rey GD. COVID-19 as an accelerator for digitalization at a German university: Establishing hybrid campuses in times of crisis. Hum Behav Emerg Technol. 2020;2: 212–216. doi: 10.1002/hbe2.201 32838228 PMC7283701

[15] van der Keylen P, Lippert N, Kunisch R, Kühlein T, Roos M. Asynchronous, digital teaching in times of COVID-19: a teaching example from general practice. GMS J Med Educ. 2020;37: Doc98. doi: 10.3205/zma001391 33364377 PMC7740025

[16] Haucke E, Walldorf J, Ludwig C, Buhtz C, Stoevesandt D, Clever K. Application of telepresence systems in teaching–transfer of an interprofessional teaching module on digital aided communication into the block training “internal medicine” during the Covid-19 pandemic. GMS J Med Educ. 2020;37: Doc84. doi: 10.3205/zma001377 33364363 PMC7740017

[17] Mikuteit M, Steffens S, Grigull L, Kühnle L, Behrends M, Schmidt R, et al. Rapid development of a digital module during the Covid 19 pandemic in undergraduate medical education of pediatrics by teachers and students. GMS J Med Educ. 2020;37: Doc66. doi: 10.3205/zma001359 33364345 PMC7740020

[18] Gottschalk M, Werwick K, Albert C, Weinert S, Schmeißer A, Stieger P, et al. Digitalization of presence events in the COVID-19 pandemia–the lecturers’ perspective. GMS J Med Educ. 2021;38: Doc30. doi: 10.3205/zma001426 33659635 PMC7899118

[19] Besse M, Wiltfang J, Belz M, Signerski-Krieger J. Implementation of digital teaching in psychiatry as consequence of COVID-19: a comparative evaluation with classroom teaching. Nervenarzt. 2021 [cited 17 Nov 2021]. doi: 10.1007/s00115-021-01081-5 33656570 PMC7927107

[20] Tolks D, Schäfer C, Raupach T, Kruse L, Sarikas A, Gerhardt-Szép S, et al. An Introduction to the Inverted/Flipped Classroom Model in Education and Advanced Training in Medicine and in the Healthcare Professions. GMS J Med Educ. 2016;33: Doc46. doi: 10.3205/zma001045 27275511 PMC4894356

[21] Dost S, Hossain A, Shehab M, Abdelwahed A, Al-Nusair L. Perceptions of medical students towards online teaching during the COVID-19 pandemic: a national cross-sectional survey of 2721 UK medical students. BMJ Open. 2020;10: e042378. doi: 10.1136/bmjopen-2020-042378 33154063 PMC7646323

[22] Zamberg I, Schiffer E, Stoermann-Chopard C. Novice and Advanced Learners’ Satisfaction and Perceptions of an e-Learning Renal Semiology Module During the COVID-19 Pandemic: Mixed Methods Study. JMIR Med Educ. 2021;7: e29216. doi: 10.2196/29216 34048357 PMC8277391

[23] Tanaka KS, Ramachandran R. Perceptions of a Remote Learning Pathology Elective for Advanced Clinical Medical Students. Academic Pathology. 2021;8: 23742895211006850. doi: 10.1177/23742895211006846 33997274 PMC8072139

[24] Suzuki T, Murayama A, Kotera Y, Bhandari D, Senoo Y, Tani Y, et al. Cross-Country Student Perceptions about Online Medical Education during the COVID-19 Pandemic. Int J Env Res Pub He. 2022;19: 2840. doi: 10.3390/ijerph19052840 35270533 PMC8910235

[25] Mukhtar K, Javed K, Arooj M, Sethi A. Advantages, Limitations and Recommendations for online learning during COVID-19 pandemic era: Pak J Med Sci. 2020;36. doi: 10.12669/pjms.36.COVID19-S4.2785 32582310 PMC7306967

[26] Ismail II, Abdelkarim A, Al-Hashel JY. Physicians’ attitude towards webinars and online education amid COVID-19 pandemic: When less is more. PLOS ONE. 2021;16: e0250241. doi: 10.1371/journal.pone.0250241 33861799 PMC8051773

[27] Guerandel A, McCarthy N, McCarthy J, Mulligan D, Lane A, Malone K. An approach to teaching psychiatry to medical students in the time of Covid-19. Ir J Psychol Med. 2021;38: 293–299. doi: 10.1017/ipm.2020.87 32611461 PMC7463153

[28] Looi JC, Bonner D, Maguire P, Finlay A, Keightley P, Parige R, et al. Flattening the curve of COVID-19 for medical education in psychiatry and addiction medicine. Australas Psychiatry. 2021;29: 31–34. doi: 10.1177/1039856220946647 32772702 PMC7424613

[29] Sahadevan S, Kurian N, Mani AM, Kishor MR, Menon V. Implementing competency‐based medical education curriculum in undergraduate psychiatric training in India: Opportunities and challenges. Asia Pac Psychiatry. 2021;13. doi: 10.1111/appy.12491 34873854

[30] Morris SG, Greenstone H, Chute R. Keeping it human: Pandemic era psychiatry teaching. Clin Teach. 2021;18: 641–649. doi: 10.1111/tct.13415 34590420 PMC8662026

[31] Baminiwatta A, Dayabandara M, De Silva J, Gadambanathan T, Ginige P, Premarathne I, et al. Perceived Impact of the COVID-19 Pandemic on Psychiatric Training Among Final-Year Medical Undergraduates in Sri Lanka: an Online Survey of Students from Eight Universities. Acad Psychiatry. 2022 [cited 29 Sep 2022]. doi: 10.1007/s40596-022-01667-4 35661338 PMC9165923

[32] Leung HTT, Ajaz A, Bruce H, Korszun A. Teaching psychiatry to medical students in the time of COVID-19: experiences from UK medical schools. BJPsych Bull. 2021; 1–10. doi: 10.1192/bjb.2021.67 34311799 PMC8326675

[33] Misamer M, Signerski-Krieger J, Bartels C, Belz M. Internal locus of control and sense of coherence decrease during the COVID-19 pandemic: A survey of students and professionals in social work. Front Psychol. 2021. doi: 10.3389/fsoc.2021.705809 34604376 PMC8479157

[34] Schiekirka S, Raupach T. A systematic review of factors influencing student ratings in undergraduate medical education course evaluations. BMC Med Educ. 2015;15: 30. doi: 10.1186/s12909-015-0311-8 25853890 PMC4391198

[35] Firmansyah F, Bandono A. Motivating Teachers During the COVID-19 Pandemic. KnE Social. 2022; 506–516. doi: 10.18502/kss.v7i10.11252

[36] Pratama H, Azman MNA, Kassymova GK, Duisenbayeva SS. The Trend in Using Online Meeting Applications for Learning During the Period of Pandemic COVID-19: A Literature Review. J Educ Innov Res. 2020;1: 58–68. doi: 10.46843/jiecr.v1i2.15

[37] Vatier C, Carrié A, Renaud M-C, Simon-Tillaux N, Hertig A, Jéru I. Lessons from the impact of COVID-19 on medical educational continuity and practices. Adv Physiol Educ. 2021;45: 390–398. doi: 10.1152/advan.00243.2020 33961515 PMC8384569

[38] Alamer A, Alharbi F. Synchronous distance teaching of radiology clerkship promotes medical students’ learning and engagement. Insights Imaging. 2021;12: 41. doi: 10.1186/s13244-021-00984-w 33765254 PMC7994478

[39] Seifert T, Becker T, Büttcher AF, Herwig N, Raupach T. Restructuring the clinical curriculum at University Medical Center Göttingen: effects of distance teaching on students’ satisfaction and learning outcome. GMS J Med Educ. 2021;38: Doc1. doi: 10.3205/zma001397 33659606 PMC7899111

[40] Reinhart A, Malzkorn B, Döing C, Beyer I, Jünger J, Bosse HM. Undergraduate medical education amid COVID-19: a qualitative analysis of enablers and barriers to acquiring competencies in distant learning using focus groups. Med Educ Online. 2021;26: 1940765. doi: 10.1080/10872981.2021.1940765 34128776 PMC8208109

[41] Bączek M, Zagańczyk-Bączek M, Szpringer M, Jaroszyński A, Wożakowska-Kapłon B. Students’ perception of online learning during the COVID-19 pandemic. Medicine. 2021;100: e24821. doi: 10.1097/MD.0000000000024821 33607848 PMC7899848

[42] Dutta S, Ambwani S, Lal H, Ram K, Mishra G, Kumar T, et al. The Satisfaction Level of Undergraduate Medical and Nursing Students Regarding Distant Preclinical and Clinical Teaching Amidst COVID-19 Across India. Adv Med Educ Pract. 2021;12: 113–122. doi: 10.2147/AMEP.S290142 33564272 PMC7866934

[43] Ibrahim NK, Al Raddadi R, AlDarmasi M, Al Ghamdi A, Gaddoury M, AlBar HM, et al. Medical students’ acceptance and perceptions of e-learning during the Covid-19 closure time in King Abdulaziz University, Jeddah. J Infect Public Health. 2021;14: 17–23. doi: 10.1016/j.jiph.2020.11.007 33341480 PMC7836241

[44] Alsoufi A, Alsuyihili A, Msherghi A, Elhadi A, Atiyah H, Ashini A, et al. Impact of the COVID-19 pandemic on medical education: Medical students’ knowledge, attitudes, and practices regarding electronic learning. PLoS One. 2020;15: e0242905. doi: 10.1371/journal.pone.0242905 33237962 PMC7688124

[45] Rios IC, Imamura M, Garcia MLB, Battistella LR. Virtual interviews between medical students and in‐patients during COVID‐19 pandemic. Med Educ. 2021;55: 663. doi: 10.1111/medu.14503 33682197 PMC8251350

