# Supplementary material for: One year of digital teaching in psychiatry as a response to the COVID-19 pandemic: Knowledge gain and content evaluation of medical students for two summer semesters in 2020 and 2021
Source: PLoS One. 2022 Oct 21;17(10):e0276660. doi: 10.1371/journal.pone.0276660 (PMC9586409; doi:10.1371/journal.pone.0276660)
Supplement: S1 Table — English translation of all 24 questionnaire items and their response scales which were used in this study. (DOCX) [file pone.0276660.s001.docx]

**S1 Table. Questionnaire items.**

| *Item formulations* | *Answer scale* |
| --- | --- |
| **(1) Knowledge gain^3^** |  |
| 1. “I am able to perform a complete psychopathological assessment.” | 6-stage^1^ |
| 2. “I know what medications are effective in treating ADHD.” |  |
| 3. “I can name the most important psychotherapeutic methods.” |  |
| 4. “I know the different types of dementia.” |  |
| 5. “I know the different classes of antidepressants with their side effects and effects.” |  |
| 6. “I know the different types of schizophrenia and their major symptoms.” |  |
| 7. “I can distinguish the different types of anxiety disorders.” |  |
| 8. “My average weekly time expenditure (incl. preparation and follow-up work, exam preparation) for the entire module was as follows in hours” | 10-stage^2^ |
| 9. “My average weekly time expenditure (incl. preparation and follow-up work, exam preparation) for the subject psychiatry in this module was as follows in hours” |  |
| **(2) Teaching contents** |  |
| 10. “The implementation of interdisciplinary teaching has been very well achieved for the subject of psychiatry in this module.” | 6-stage^1^ |
| 11. “In this module, independent study of the learning objectives of the subject of psychiatry was encouraged.” |  |
| 12. “In terms of my professional future, I rate my knowledge gain in psychiatry in this module as very high.” |  |
| 13. “I was very satisfied with the basic structure of the module (structure, teaching formats, schedule) related to the subject of psychiatry.” |  |
| 14. “I was very satisfied with the practical implementation of teaching related to the subject of psychiatry in this module (absence rate, lecturer punctuality).” |  |
| 15. “This module should be continued as is with respect to the subject of psychiatry.” |  |
| 16. “The psychiatry lectures in this module contributed significantly to my learning progress.” |  |
| 17. “The psychiatry seminars in this module contributed significantly to my learning progress.” |  |
| **(3) Subjective advantages of digital teaching^4^** |  |
| 18. “My knowledge gain in the subject of psychiatry is greater with digital teaching than in classroom teaching.” | 6-stage^1^ |
| 19. “For the future, I would like to have predominantly digital courses in psychiatry.” |  |
| 20. “I estimate my time commitment for the subject of psychiatry to be higher with digital teaching than with classroom teaching.” |  |
| 21. “Compared to classroom teaching, digital teaching has prepared me better for the written state exam in psychiatry.” |  |
| 22. “Compared to classroom teaching, digital teaching prepares me better for my future medical profession.” |  |
| 23. “The COVID-19 pandemic has reduced my learning progress.” | 6-stage^1^ |
| 24. “The COVID-19 pandemic has increased my general stress level (psychological well-being, family, etc.).” |  |

*Notes.* English translation of items. Items were answered on a ^1^scale from 1 to 6 (1 = “fully applies” to 6 = “does not apply”), except for control items no. 8 & 9 which were answered on a 10-stage ^2^scale from “5 hours” up to “50” hours. ^3^*Knowledge gain*: Items no. 1 to 7 were answered twice: (1) *retrospective* (pre-teaching) and (2) *current state* (post-teaching). ^4^*Subjective advantages of digital teaching*: Two control items were added to measure the general effects of the COVID-19 pandemic on learning progress (no. 23) and general stress level (no. 24)
